# The Impact of Sex Work on Women’s Personal Romantic Relationships and the Mental Separation of Their Work and Personal Lives: A Mixed-Methods Study

**DOI:** 10.1371/journal.pone.0141575

**Published:** 2015-10-30

**Authors:** Clare Bellhouse, Susan Crebbin, Christopher K. Fairley, Jade E. Bilardi

**Affiliations:** 1 Melbourne Sexual Health Centre, Alfred Health, Melbourne, Victoria, Australia; 2 School of Health Science and Psychology, Federation University, Ballarat, Victoria, Australia; 3 Central Clinical School, Monash University, Melbourne, Victoria, Australia; 4 Nexus Primary Health, Melbourne, Victoria, Australia; Örebro University, SWEDEN

## Abstract

**Background:**

Very limited research has been undertaken on sex workers’ personal romantic relationships and the impact the nature of their work has on their relationships. This exploratory study aimed to explore the impact sex work has on women’s personal romantic relationships and the use of mental separation as a coping mechanism to balance the two aspects of their lives.

**Methods:**

Fifty-five women working in the indoor sex industry in Melbourne, Australia, were recruited to complete a self-report questionnaire about various aspects of their work, including the impact of sex work on their personal relationships. Questionnaires were completed anonymously and included both closed and open-ended questions. A further six women were interviewed to ‘member check’ the accuracy of the questionnaire findings.

**Results:**

Most women (78%) reported that, overall, sex work affected their personal romantic relationships in predominantly negative ways, mainly relating to issues stemming from lying, trust, guilt and jealousy. A small number of women reported positive impacts from sex work including improved sexual self-esteem and confidence. Just under half of women were in a relationship at the time of the study and, of these, 51% reported their partner was aware of the nature of their work. Seventy-seven percent of single women chose to remain single due to the nature of their work. Many women used mental separation as a coping mechanism to manage the tensions between sex work and their personal relationships. Member checking validated the accuracy of the questionnaire data.

**Conclusion:**

This exploratory study identified a number of ways in which sex work impacts negatively on women’s personal romantic relationships. The findings of this study support the need for further studies to be undertaken to determine if the findings are reflected in a larger, more representative sample of Australian sex workers and should be considered in the context of any future intervention and support programs aimed at addressing the tensions sex workers experience between their work and personal relationships. Greater public awareness and education programs aimed at addressing the negative stigma associated with the sex industry may go some way towards easing the issues faced by women in their personal relationships.

## Introduction

Sex work involves one or more services where sex is exchanged for money or goods [1]. Sex workers however are not a homogenous group [2, 3]. Street sex workers are generally illegal workers who meet clients on the street and service them in alleys, or the clients’ cars, whereas indoor sex workers are employed to work in brothels, massage parlours or as call girls [4]. Past research has shown both street sex workers and indoor sex workers have commonly experienced high levels of abuse in childhood and adulthood [5–10]. While rates of abuse and trauma are lower for indoor sex workers than street sex workers, they are still higher than the general population [10–13].

Sex workers commonly face significant stigma related barriers regardless of where they work, due to their perceived violation of gendered norms through sex with multiple partners and strangers, taking sexual initiative and control, inciting male desires, and receiving fees for sex [14–16]. Stigma can be external and enacted through discrimination by others, or an internalised sense of shame [15], and is generally accompanied by an intense fear of others ‘finding out’ about their work due to the stigma associated with it [14, 17–19].

In Australia, legislation surrounding sex work varies by state and territory and in a number of states/territories some forms of sex work are legal [20]. In Victoria, under the Prostitution Control Act 1994, indoor sex work in a licensed brothel, escort agency or private setting is legal, however street based sex work remains illegal [21]. As part of legislature, indoor sex workers cannot knowingly work with a sexually transmitted infection (STI) and are required to have three monthly STI testing and provide a certificate to work [22].

Information from the AIDS Council of NSW (a state in Australia) suggests approximately 20,000 legal and illegal sex workers are working in the Australian sex industry at any one time [20]. Most sex workers in Australia predominantly work in the legal indoor sex industry, with estimates of between 2%-10% of street based workers in the industry [20, 23, 24]. It is difficult to provide estimates of the number of sex workers due to the transient nature of sex work and sex workers reluctance to report working in the sex industry. This is often due to aspects which remain criminalised which may result in them incriminating themselves or making themselves a target of abuse (20). The stigma surrounding the industry also means they are often reluctant to disclose their work even with family and friends, and the knowledge that in Australia once registered, their name will remain listed in a sex work database regardless of whether they are still working in the industry [20].

While there are some commonalities between street sex workers and indoor sex workers, generally there are vastly different issues associated with their work and substantially different conditions in which it takes place [9, 10, 18]. Indoor sex workers are far less likely to report injecting drug use or issues around poor health compared to outdoor sex workers (20). They are also less likely to report concerns around personal safety or to experience work related violence compared to outdoor workers due to regulations and controls in place in the legal sex industry [20]. Indoor sex workers are also more likely to view their work as a career than a transient job and remain in the industry long-term and tend to express different concerns in relation to their work including problems surrounding their personal relationships [18].

While considerable research has been conducted on street sex workers, there is considerably less data on indoor sex workers. The majority of past research on indoor sex workers has related to condom use and physical health. Past research has found that many sex workers use condoms with clients but are less likely to in their personal lives [19, 25–29]. The absence of condoms appears to signify security, intimacy and trust between sex workers and their personal partners. Perceived intimacy is the strongest predictor of non-condom use, with condoms serving as an emotional, physical and symbolic barrier between sex workers work and personal lives [27, 30, 31].

In a study by Warr and Pyett [31] of condom use in female indoor and street sex workers’ personal romantic relationships in Victoria, Australia, researchers found that almost all 24 women did not use condoms in their private relationships in order to maintain a distinction between sex with clients and sex with private partners. All of the women reported tensions associated with working in the sex industry and having a private sexual relationship including issues of jealousy, resentment, disapproval and disrespect from partners due to the nature of their work. A recent study by Bilardi et al. [25] based in Melbourne, Australia, examined female sex workers’ job satisfaction but also briefly noted that women working in the sex industry reported problems in their personal relationships which stemmed from their work. Most participants reported that sex work interfered with their romantic relationships adversely due to issues of jealousy, guilt and safe sex practices. Seventy-five percent of women stated that the job made it too difficult to sustain a relationship and 80% reported that sex work interfered with romantic relationships.

Previous studies that have touched on sex workers personal relationships as part of the broader study have also found sex work negatively affects personal relationships. In a study by Sanders [32], which examined sex work from the perspective of risk management in England, it was found that negative emotions generated by the commodification of women’s bodies through sex work affected their social identities and relationships, with women struggling to separate sex at work with sex for pleasure. In a further study by Rossler et al. [33], examining the mental health of women in the sex industry in Switzerland, women stated relationships commonly failed because their partners could not separate out sex at work with sex at home even though the women themselves could.

To cope with these issues, sex workers commonly adopt behaviours to separate their work and personal lives [17, 19, 29, 32]. Work/family border theory proposed by Clark [34] argues that work and family influence each other. Work and home lives differ in terms of purpose as well as culture and have specific patterns of attitudes and behaviour for each. Some level of integration is necessary to balance the two spheres of an individual’s life, but the degree to which this occurs varies between people. According to work/family border theory, borders are lines of segregation between domains, defining where a person’s behaviours begin and end. A physical border defines where these take place, temporal borders define when the behaviours take place and psychological borders are defined by the individual, dictating when behaviours, thinking patterns and emotions are appropriate for each. The more flexible a border is, the more an individual can think about work while at home and home while at work. When domains are very different it can be more difficult to juggle the conflicting demands and an individual can experience confusion about their identity and purpose [34].

In a study by Wolffers et al. [29] on female sex workers in Indonesia, the majority of who were married or had previously been married, two important ways sex workers kept their work and personal lives separate were living far away from work and changing their dress and make-up. Another important aspect was maintaining emotional distance at work while being emotionally involved at home. Other strategies sex workers commonly use to cope with the demands of sex work are taking regular breaks at work, physical boundaries between work and home, keeping to time during consults, hiding appearance and avoiding emotional relationships with clients [17, 19]. This can however, lead to issues with dissociation and denial and affect women’s mental health [35, 36]. Similarly, Sanders [19] found indoor sex workers constructed a manufactured identity in order to maintain a sense of self by limiting certain feelings to work, and certain feelings to their personal lives. Women had certain rituals surrounding clothing, behaviour and appearance to separate their identities, with some women even referring to their work persona in the third person.

The romantic relationships of indoor sex workers, outside of work, has not been studied extensively despite being raised as a concern by many women in the sex industry [25, 29, 37]. It is likely that the nature of sex work impacts on many areas of women’s personal relationships. It could be argued that psychologically, activities at work and home are very different for sex workers and therefore strong borders between the two would be required in order for a person to cope with the very differing demands. This study developed from findings of an earlier study by Bilardi et al. [25] which found that female sex workers struggled with the tensions between sex work and their personal romantic relationships. The aim of this study was to explore the impact sex work has on women’s personal romantic relationships and the use of mental separation as a coping mechanism to balance the two aspects of their lives.

## Method

This exploratory study allowed for preliminary investigation in an area in which very limited data is currently available. Exploratory studies aims to explore the research questions, gain greater understanding of an issue and lay the groundwork for further investigation into the area of study [38]. A mixed-methods approach was used as it allowed for the use of multiple methods to explore, identify and confirm findings within the study. Mixed method studies commonly employ both qualitative and quantitative approaches to allow for greater breadth and depth of understanding and are useful in exploratory design studies [39].

### Ethics statement

Ethical approval for this study was granted by the Alfred Hospital Ethics Committee, Victoria, Australia, Application Number 244/14 on the 12^th^ June 2014.

### Participants

To be eligible for the study women had to be over the age of 18, have a good understanding of English, and work in a licensed brothel, massage parlour or as a private escort in Victoria, Australia. Participants were recruited between June and August 2014 from the Melbourne Sexual Health Centre (MSHC), the largest sexual health clinic in Victoria, Australia, where they attended for their three monthly check up and certificate to work. Eligible participants were identified through the Computerised Patient Management System (CPMS) and approached by nurses during the triage process.

### Data collection

#### Anonymous questionnaire

Women were asked to complete a broader 54 item self-report questionnaire which asked about sex workers’ relationships, work characteristics, rates of abuse, personality type, levels of mental separation between sex work and personal relationships, self-esteem levels, resilience levels, perceived levels of social support from friends, family and the wider community, and intimacy with their partner. This study reports only on the 31 questions relating specifically to work characteristics, personal relationships, rates of abuse, condom use, and the levels of mental separation between sex work and personal relationships.

Of the 31 questions, 25 questions relating to sex work, relationships and condom use were taken from the previous study of sex workers’ job satisfaction based at MSHC by Bilardi et al. [25] Of the remaining six questions, one was derived from a life hassles scale [40] used to measure rates of abuse, with one item relating to childhood sexual abuse removed from the scale as it was deemed too confronting for the purposes of this survey. Three questions measuring the separation of work and personal relationships were developed based on a scale of work-family conflict [41]. An additional two questions were developed by study investigators and related specifically to sex work and personal relationships. Questions included both closed and open ended responses.

#### Member checking

Following completion of the questionnaire data collection and analysis, a further six women were independently recruited to take part in semi-structured qualitative interviews to ‘member check’ the study findings. Member checking can be undertaken for a variety of reasons, including as a means of validating study findings and ensuring the credibility of results [42]. Participants were first asked to describe their background in the sex industry before they were verbally presented with the major findings of the study and asked to comment on whether the findings reflected their personal and broader experience of working in the indoor sex industry.

### Recruitment

Women were opportunistically recruited to the study during a routine three monthly clinical appointment for sexually transmitted infection testing to obtain their certificate to work. Women were identified through CPMS. During the consultation a nurse briefly explained the study to eligible women and invited them to participate. Women interested in participating were offered a plain language statement and questionnaire at the end of their consultation and given the option to complete the questionnaire privately onsite or complete the questionnaire off-site and return it in a reply paid envelope. The questionnaire was anonymous with no identifying information collected. Women involved in the member checking interviews were recruited by the same method and interviewed either face to face or by telephone, depending on their preference.

### Data analysis

Questionnaires were entered into SPSS and analysed using descriptive and frequency analysis. Open-ended questions were transcribed verbatim and thematic analysis applied. Thematic analysis is a method of identifying, analysing and reporting patterns in qualitative data, which are commonly referred to as themes, to organise the data and convey important and relevant meanings [43]. Open-ended responses were firstly read and re-read by CB to identify the major themes and categories arising from the data. Themes were developed based on relevant background literature, questions derived from the aims of the study and issues raised by women. Once identified, themes and categories were coded and text responses grouped according to similarities and differences. Responses were re-read again by CB to further revise, refine and confirm categories. To ensure consistency and reliability of data analysis, two secondary researchers (JB and SC) examined a subset of qualitative questionnaire responses to cross check themes and categories. No further themes or differences in interpretations were identified by either secondary researcher.

Member checking interviews were digitally recorded, transcribed verbatim and the same thematic analysis process applied.

## Results

A total of 55 women completed the questionnaire. In addition, one woman started and returned the questionnaire but only completed the initial demographic questions and her results were therefore not included in the study. For pragmatic reasons and due to the anonymous nature of the questionnaire we were unable to keep a record of all the women who accepted a questionnaire and did not complete and return it, or the reasons for non-completion. Table 1 summarises the demographic characteristics of participants. All 55 participants had been in a relationship at some point in their lives. Over half of the women in the study were single at the time of completing the questionnaire. Of the women who were in a relationship, just under half were married or living with their partner. The relationship demographics of the sample are summarised in Table 2.

**Table 1:** Participant characteristics (N = 55).

	N (%)* or Median [Range]
Age	29 [20–48]
Born in Australia	30 (55)
Currently studying	24 (44)
*Education level completed to date*	
Less than Year 12	8 (16)
Year 12	15 (29)
Trade/certificate	7 (14)
Undergraduate university degree	17 (33)
Postgraduate university degree	4 (8)
*Type of establishment*	
Brothel	36 (71)
Massage parlour	8 (16)
Private/escort	7 (14)
Length of time in sex work (months)	27 [1–336]
Hours worked per week	25 [1–80]
Worked on weekends	38 (64)
*Top reasons women entered sex work*	
Needed money	46 (90)
Attracted to flexible working hours	46 (85)
Particular goal in mind	36 (68)
Physically abused in the past	34 (65)
Sexually abused in the past	31 (60)

*Numbers may not add up to 55 due to missing data. Percentages have been calculated using valid cases. Percentages have been rounded up to 0 decimal points.

**Table 2:** Participants relationship characteristics (N = 55).

	N (%)* or Median [Range]
*Relationship status*	
Married/living with partner	11 (20)
In relationship but not living with partner	14 (26)
Single	30 (55)
*Partner gender*	
Male	47 (90)
Female	4 (8)
Male and female partner	1 (2)
Duration of current relationship (months)	24 [2–168]
Partner employed	35 (59)
*Top reasons women were not in a relationship*	
Choose to be single	26 (77)
Not met someone suitable	24 (71)
Job makes it too difficult	19 (56)
Do not trust men	19 (56)
Time since last relationship (months) for single women	18 [6–168]
Duration of past relationship (months) for single women	24 [2–144]
Partner aware of work	25 (51)
*Condom use*	
Always wear condoms for sex with clients	53 (96)
Always wear condoms for sex with regular partners	13 (27)
Always wear condoms for sex with casual partners	27 (60)

*Numbers may not add up to 55 due to missing data. Percentages have been calculated using valid cases. Percentages have been rounded up to 0 decimal points.

The majority of the women who were in relationships believed that overall, sex work affected their romantic relationships (78%) in mainly negative ways.

### Negative impact of sex work on relationships—Women in relationships

The main ways in which sex work negatively impacted on women in relationships were around issues of dishonesty and distrust, jealousy, stigma and pragmatic issues. Table 3 provides further quotes from women around these issues.

**Table 3:** Issues single women and women in relationships face in their personal relationships as a result of sex work.

Women in Relationships	Single Women
*Dishonesty and distrust*	*Dishonesty and distrust*
I have trust issues—are they having sex with others…? (Participant 27).	I don’t expect anyone to be ok with me working. I wouldn’t if the shoe was on the other foot. So it’s lying and deceiving constantly (Participant 5).
I’m concerned that he will reject or judge me. It’s also increased his suspicions about my sexual activity (Participant 44)	I can't always be honest as to what has me stressed and tired (Participant 14).
	I don’t trust men anymore. I enjoy sex however don’t have time to meet decent men outside of work (Participant 38).
*Jealousy*	*Discomfort*
Depends on the man. My ex husband (sic) didn’t really care so that showed his level of commitment to me. My current partner hates anyone else touching me and worries I may get hurt (Participant 9).	If I was to get a partner, I don't know how they would react to my work (Participant 4)
*Stigma and sex work*	*Stigma and sex work*
Romantic interests are sometimes discouraged by the nature of the work, holding beliefs that stigmatise the industry (sic) (Participant 16).	Not many people understand the nature of this work. If someone wants to be in a relationship with me, knowing what I do, they seem to assume I have low moral standards (Participant 27).
Most males couldn't or wouldn't cope with the situation. The sex industry is still overly stigmatised (Participant 25).	It's just work. I don't see how different it is to any other job. The only problem I have is how stigmatised it is (Participant 4).
There is a gap between the nature of my job and the public perception (Participant 15).	As I am dating again, I find that many men and women carry the notion that sex workers are drug addicts/desperate/diseased. I find it is easier not to discuss work until I discover the person's notions around the industry. If they are negative I stop dating them (Participant 51).
*Pragmatic issues*	*Wrong type of partner*
After working, you can be so sore/sensitive to go home and have sex with your partner, which can cause problems (Participant 50).	Now I only want to be in a relationship with someone who wouldn't want me to work, because they wouldn't want to share me with anyone, not because they have a problem with my work, therefore while I work I can't date (Participant 48).
Energy levels and sex life sometimes (Participant 21).	I couldn’t lie about my work and I couldn’t respect someone who allowed me to do sex work (Participant 23).
	I would never enter into a relationship whilst in the sex industry because I don't think it is the person I want to be. And/or I wouldn't want anyone to know let alone my partner, and I wouldn't want anyone to be ok with me doing that (Participant 49).

#### Problems in general

Some women commented that sex work caused problems in their relationships but did not elaborate further.

*I have a lot of problems in my relationship because of my work… I just don't want to cause any more problems*.

(Participant 31)

*The job doesn't help when in a relationship*. *It's much easier to be single*, *but I am human*, *I have feelings*. *When work is not brought up it is usually fine*, *but I do feel bad when I think about what they have to put up with*.

(Participant 12)

#### Dishonesty and distrust

Of the women in relationships, only half had told their partners they were working in the sex industry. Women who had not told their partners about their work commonly expressed concerns about lying to their partners and the guilt this caused them to feel, which in turn raised issues of trust. Some women who were lying to their partners about the nature of their work questioned whether their partners might also be deceiving them.

*I have trusting problems*, *I feel guilty*. *All the time I feel like I am a bad person*.

(Participant 47)

Women were commonly worried about their partner finding out about their work or thinking they were being unfaithful.

*All the lying*, *I have to make up excuses and he is very suspicious*. *We always fight about it*.

(Participant 52)

For these women, not telling their partners about their work led to questioning about their faithfulness.

#### Jealousy

For women who had told their partners about their work, the impact of sex work on their relationships was largely determined by how their partners reacted when they found out and how they felt about them doing sex work. The majority of women reported that being honest with their partners about their work had impacted negatively on their relationship rather than positively. Problems often arose when partners had issues with the nature of their work, and experienced jealousy, resulting in arguments.

*My relationship before this*, *the guy found it very hard to deal with*. *Would call while at work so I wouldn’t do intros* (sic), *tried to accept but was hard*, *caused many fights*. *He didn’t see it as just a job*.

(Participant 12)

#### Stigma and sex work

The stigma associated with sex work in the wider community was a major barrier for most women in their relationships, causing difficulties with the level of support and understanding they received from their partners.

*It is not the fact that I am a sex worker but the fact that stigma is attached to the work*, *that can cause issues*.

(Participant 24)

#### Pragmatic issues

Other issues in relationships were more pragmatic, with many women reporting that after having to have sex with clients at work all day they were tired and did not want to come home and have sex with their partner.

*Too tired from work and sometimes making love feels like being with a client*.

(Participant 11)

#### Positive impact of sex work on relationships—Women in relationships

While most women reported negative impacts on their relationships from sex work, a few felt that sex work had positively impacted on their relationships. These women felt that sex work had enabled them to experience deeper intimacy with their partners and that sex work improved their private sex life as well as their self-esteem and confidence.

*We are closer because I need to be more honest about my sexual energy and needs*. *It has also proven he is not a possessive or sexist man which is important to me*. *And I’m more assertive and confident sexually*.

(Participant 8)

*Being a dominatrix has given me so much confidence and makes me proud to do the work I do*. *I’m more sensitive to others and life is better overall*.

(Participant 20)

Women who reported positive impacts on their relationships from sex work tended to take a holistic view of sex work, regarding it as an important part of their life and who they were. These women were less inclined to feel the need to separate their work and home lives, which in turn impacted positively on their personal lives and relationships.

*I don't separate it too much*. *It is my life and all parts are important*. *I am also lucky to have supportive SW* (sex worker) *and non-SW* (sic) *friends and family*.

(Participant 20)

Some women similarly felt that their profession was better understood, and it was easier on their relationship, if they were dating ex-clients who had an understanding of the nature of their work due to their prior experience of sex worker services.

*I met my current partner through work*, *he was a client*. *That in some ways has made it easier to negotiate being a sex worker because he knows what I do*.

(Participant 20)

### Single women

Over half of the women in the study were single, mainly out of choice, and mostly due to the nature of their work. Some women reported that generally the nature of their work was not conducive to having a relationship, however they did not elaborate further.

*I can’t have a relationship whilst working*, *I only work when I’m single*.

(Participant 10)

#### Discomfort

More commonly women reported that they chose to remain single while doing sex work either because they were not comfortable with being in a relationship while working in the sex industry or because they felt that partners would not be comfortable with the nature of their work.

*If he doesn’t know there are too many lies and you fall out of love*, *if he does know then the* (sic) *partner would be uncomfortable*.

(Participant 13)

#### Wrong type of partner

Interestingly, quite a few women specifically commented that they would not want to be with someone who was comfortable with them being a sex worker.

*Even if your partner said he was ok with it*, *I don’t think he would be or should be*, *you would always be thinking in the back of your head ‘why he is ok with it* *?’*.

(Participant 54)

Generally, these women assumed that while they were working it would be better to stay single because the sort of partner they would want to be with was not the type that would want a partner doing sex work.

#### Dishonesty

Other women reported that they felt the need to lie to many people in their lives about the nature of their work and they did not want to lie to a sexual partner, which is why they preferred to stay single while working in the sex industry.

*I don’t feel I can be intimate when my ‘work’ is a lie—I’m affectively lying to everyone and this is hard*.

(Participant 23)

Women commonly felt they could not be honest about the nature of their work and this created barriers with relationships and intimacy.

#### Stigma and sex work

Single women also struggled to be honest about the nature of their work due to the stigma attached to the sex industry. Single women often reported that potential partners did not understand the true nature of their work and the stigma associated with it caused many partners to react negatively.

*There is a gap between the nature of my job and the public perception*.

(Participant 15)

#### Distrust

A number of women also spoke of an inability to trust men which developed either early in their lives as a result of physical or sexual abuse or as a consequence of sex work, impacting heavily on their desire to have a relationship.

*Because of all the nice and lovely men I have met through work (not the pricks) I no longer trust men to be faithful*.

(Participant 14)

Trust had become a huge issue for some women because of their exposure to men as clients.

*I now think about 85% of men are psychopaths*. *I am reclusive and have been hurt/suffered abuse…it’s more about knowing other people’s limitations and therefore being aware so as to protect myself from any hurt*.

(Participant 14)

*It affects trust*, *it affects how you see and feel about sex*, *it affects your whole personality/life if you let it*.

(Participant 37)

Three sex workers in particular reported that their work had a substantial impact on all facets of their lives. Sex work had become something that defined their whole lives and these women seemed to be more desperate to leave the industry altogether.

#### Relationship status not due to sex work

While many women felt their work kept them from having relationships, a minority reported they were not single because of their work nor did their work have a major impact on their relationships.

*Being a sex worker doesn’t effect* (sic) *my view of relationships*. *If I was to meet someone and there was a chance of anything*, *I would tell them what I do*. *Their reaction to it is their business*.

(Participant 4)

*I don’t believe working should affect intimacy and in future my men will accept it*.

(Participant 43)

These women expressed a desire to be in a relationship, be honest about their work and find a partner who would be comfortable and accept their work. If a partner however, could not accept the nature of their work, they felt this would be their partner’s problem and not theirs to be concerned about.

### Separation as a coping mechanism

About half of women, either single or in a relationship, spoke about the need to maintain a distinction between their work and personal lives, some however, found this easier to do than others. Over half of women (53%) reported they found it either *quite* or *very difficult* (measured on a 10-point Likert scale) to mentally separate their work life from their personal life. This was often because they felt they were deceiving people in their personal lives.

*If problems occur at work*, *it may be hard to hide them in your personal life*. *It’s hard to always keep the lie up*. *It’s difficult to answer the question*, *‘how was your day’*.

(Participant 6)

*It has become harder* (to separate), *this is because it kills me to lie and as an older sister I wish I could set a more responsible and steady example*.

(Participant 14)

Most women separated their work life from their home life, mainly to try and limit the impact of their work life on their personal life.

*I have 2 personas who* (sic) *live comfortably side by side*.

(Participant 23)

*I'm pretty good at maintaining it all separately*. *However*, *I am on anti-depressants which helps a lot*.

(Participant 6)

*I switch off when I am not at work*.

(Participant 33)

Of the women, a few reported ways in which they separated their sex work from their personal lives including one sex worker who reported that to keep her work life and personal life separate she did not spend time with other sex workers outside of work.

*I keep it separate*, *I do not hang out with other workers*.

(Participant 41)

A number of other women reported that condom use was a way in which they separated sex at work with sex at home. Women generally used condoms with their clients but not with their personal partners.

*I sleep with my husband without protection but always practice safe sex with clients*.

(Participant 11)

*Never with my former partner as he'd had a vasectomy and we were both checked out and tested*.

(Participant 10)

While trying to separate their two lives may have been useful for some women, others found that trying to separate their work and home life made things more difficult and isolating.

*I find it isolating and stressful to not be able to discuss work at home or with friends*.

(Participant 44)

It was particularly difficult for women in committed personal relationships.

*It used to be quite easy* (to separate) *but I am in love with my current partner and this makes it very hard*.

(Participant 9)

*Sometimes making love feels like being with a client*.

(Participant 11)

### Member checking

Overall, women who member checked the questionnaire results agreed with the findings of the study. Tables 4 and 5 provide an overview of their experiences in the sex industry and examples of the impact sex work has on their personal relationships and their use of mental separation as a coping mechanism. The 55 women’s experiences largely reflected the six women’s own experiences or those of women they knew working in the indoor sex industry. As one woman exclaimed when reading a statement relating to women wanting a partner who was not comfortable with them doing sex work, ‘*Yes*! *Yes exactly*! [nodding]’ The women interviewed represented a diverse group of indoor sex workers and as a result, women were not always able to personally relate to all of the quotes and findings presented to them. However they often stated that while certain aspects did not necessarily reflect their own personal experience, they did reflect the experiences of other sex workers they knew, ‘…*not for me personally but I can understand where these girls are coming from*’. The main difference found between the experiences of the 55 sex workers who completed the questionnaire and the six women interviewed for member checking was that the member checking women were more likely to focus on both the positive and negative effects of sex work on their personal lives and relationships. Women who completed the questionnaire were more likely to report on the negative effects.

**Table 4:** Member checking—Single Women.

Annie	Akina
Annie began working in the sex industry due to negative dating experiences, deciding it would be better to work as a sex worker and get paid to ‘have fun’ without the hassles of dating. She had largely positive experiences from her sex work and felt that in order to have a relationship she would have to find someone similarly ‘like-minded’ who was comfortable with her work. She did, however, believe that the stigma surrounding sex work was an issue providing an example of a friend who did not know she worked in the sex industry discussing the topic with her:	Akina was born overseas and began working as a sex worker to save money to travel. When she arrived in Australia she once again began working in the sex industry but had not told any of her friends at home what she did for a living. She explained that sex work made her feel guilty and equated it to cheating. She thought there was a lot of stigma surrounding sex work but that this was worse in her country of birth than in Australia. She did not want to be with a man who would let her do sex work
*She was like*, *‘Oh my god*, *you’re the nicest person…’ but then again I wonder how she would feel*, *if I told her what I do*, *how she would then view me*? *You know she was saying that*, *you know ‘Prostitutes and strippers they wreck marriages’*.	…*maybe I’m not really happy with the guy who can say it’s okay but it doesn’t really… if I really love him*, *I don’t know I’ve never really had a guy like that*. *I think I’ll be happier if the guy cares about me and then don’t do it*. *I want him to say no*.’
Annie did not feel the need to separate her work and personal lives and instead viewed her work as integrated with the rest of her life.	Akina deliberately kept her personal life separate from her work life.
*…like I say to my clients ‘You guys are all like my boyfriends without the headache’*.	…*some of my friends who’s doing sex work*, *they gave the clients their number and sometimes they go to dinner or something*, *like ohhhh my god I can’t do it (laughs)*, *yes*, *separate*, *I never want them to come into my private life*.

**Table 5:** Member checking—Women in Relationships.

**Samantha**	**Charlotte**
Samantha began sex work due to financial difficulties following the breakdown of her marriage, at which time she had mental health issues at the time and an unsupportive partner. At the time she was unemployed, homeless, did not have custody of her two children and needed money quickly to get back on her feet financially. She reported previously having a nine month relationship with a partner who wanted her to stop sex work and said he would support her financially. She stopped sex work but the relationship broke down and she re-started sex work to support herself. She did not want to remain in the sex industry but needed the money. The man she was currently dating was more supportive of her work in the sex industry.	Charlotte and her partner have an open relationship and began sex work after they began getting paid to attend ‘swingers’ parties every few months that they had already been attending as a couple. Following this Charlotte was asked to do threesomes which she enjoyed however she did not want this as her only job and so she started working privately as an escort. She reported she had been reluctant to do escort work very often though due to safety concerns and ended up working in a parlour instead. She felt that sex work had some important positive impacts on her life.
*…he believes he doesn’t have a right to tell me not to do it or that he doesn’t like me doing it*. *He doesn’t believe he has the right to say that*.	*I was always very self-critical*. *And it wasn’t until I started swinging that I realised actually people do find me desirable and I guess that’s what pushed me into sex work*. *I do totally agree though that I feel empowered*, *like I hold the cards*.
She also reported that stigma surrounding the sex industry was a huge problem.	Charlotte has kept her work secret from others mainly because she wants to protect her young children from bullying and the stigma associated with sex work. Charlotte kept some sex acts separate from work and home.
*…and you know it’s a stigma*, *similar stigma with mental health and the issues that people face with having a mental illness in society*. *It’s not accepted*.	*Like I just keep between me and him*. *Like if I do it all the time at work it’s no longer special… I promise him I won’t do it to anyone else*.
Samantha felt that she had a separate persona she put on at work to separate it from her personal life.	
*I can be professional and I can be somebody else and you know walking into an acting job and playing that role and at the end of the day I take off the clothes and the make-up and I put on*, *you know*, *and I’m just me then*.	
**Lauren**	**Anna**
Lauren had worked as a receptionist in a brothel for years before deciding to try sex work for a few months to gain an insight into the experiences of sex workers, ‘*on the other side of the desk*’. She continued to do sex work to supplement her income after losing her reception job, in conjunction with a further desk job. She met her partner through sex work but as the relationship became more serious he was not comfortable with her working. At the same time she also became pregnant to a client. She quit sex work but then lost her desk job and had to go back to sex work again but did not tell her partner.	Anna began sex work as she had been sleeping with a number of partners but found this ‘*disappointing*’ and ‘*unfulfilling*’ and wanted to have more sexually adventurous experiences. She felt sex was something she was very good at and would like to try it in a safe and controlled environment, like a brothel. Her partner was living interstate and she had not told him about her work as she anticipated that he would think it was ‘*wrong*’. She felt that she may also not be comfortable if he was doing sex work and therefore he would not be with her doing it.
*…and he would drop me off sometimes and I would literally walk through the door*, *go down to the underground carpark*, *sit in the underground carpark for 10 minutes knowing that the car*, *he’d be gone by then*, *and then get out of there and go [to work]*.	*I just think ultimately we just have different values*. *I don’t know if I would be okay with it if he was having sex with people for money*, *but just because I am so sure in how I feel about my work*, *and how it’s work*, *I feel really confident in knowing that*, *like they’re separate but like he would never understand that*.
She found it very difficult to keep lying to her partner.	She did not understand the stigma associated with sex work in the wider community.
She separated her work and home life by developing a different persona with a different name.	*…she [author of ‘Secret Diary of a Call Girl] says this thing about how society is ok about like a man and a woman meeting each other in a bar and going home to have sex and how it’s totally not ok if an agency has arranged that and checked that the guy is like ok and money changes hands and I really feel like it is*, *it’s just sex*.
*…I used to come into work and think ‘Oh great*! I’m *Sally* and *Sally* doesn’t have to think about how she had a fight with her sister or uni’s [sic] hard or there’s a mountain of bills at home and her Mum is stressing. *She can be happy and fine*.	Anna kept things separate by creating boundaries around the location of her work and personal lives.
	*…work happens at work and I leave and just go home and eat a salad*, *it’s not*, *there’s no*, *yeah I feel really separate*.

## Discussion

Research regarding the impact of sex work on romantic relationships is very limited yet our findings suggest that the nature of sex work affects women’s romantic relationships significantly. Just under half of women were in a relationship at the time of completing the questionnaire, and of these women, just over half reported their partners were not aware they were working in the sex industry. For the majority of women this resulted in feelings of guilt from lying and a fear of their partners finding out, which in turn caused trust issues for both partners, with some women’s partners becoming suspicious about their sex lives and faithfulness. The majority of women who had told their partners they were working in the sex industry experienced largely negative impacts around jealousy and misunderstanding due to the stigma associated with the sex industry. Interestingly, the difficulties women in relationships reported due to the nature of their work were the same issues or reasons why many women chose to remain single while employed in sex work. A few women reported positive impacts of working in the sex industry and being in a relationship, including an improved sex life, higher levels of intimacy with their partner and improved self-esteem and confidence. Women in this study commonly experienced issues negotiating the borders between their work and personal relationships, especially in regards to a negative ‘spill over’ of emotions from work into their home lives and relationships. Over half of women reported they found it difficult to mentally separate their work life from their personal life, using mechanisms such as not socialising with other sex workers or using condoms with clients but not with romantic partners to separate the two spheres.

### Sex work and relationships

The findings from this study support and extend previous findings [25, 33, 37] which have also found that women working in the sex industry commonly report negative impacts on their relationships as a result of their work due to issues around lying, trust and feelings of guilt. In a study by Warr and Pyett [37] of condom use among women working in the sex industry in Australia, women in relationships commonly experienced similar negative impacts due to the nature of their work. Past research has shown that it is not uncommon for couples in other occupations to also experience negatives issues associated with suspicion, jealousy and questions of faithfulness [44]. These issues commonly result if violations of trust and loyalty occur, which are thought to be integral to relationship satisfaction. As couples in other occupations commonly experience these negative issues, it is likely that these problems would only be heightened in sex workers’ personal relationships given the nature of their work and the stigma associated with the sex industry.

As previous studies have also found [14–18], stigma was a major barrier in sex workers personal romantic relationships, with women commonly reporting that partners misunderstood the true nature of their work due to negative stigma surrounding the sex industry, leading to significant problems in their relationships. Sex workers are stigmatised as ‘whores’, damaged women of loose moral values and character whose practices lie outside the boundaries of ‘normal’ prescribed sexual behaviour, resulting in stigma characterised by fear, disapproval, rejection, and shame [45]. A study by Scrambler [14] of female sex workers in London, England, found that women experienced ‘whore’ stigma from the wider community, attributing blame and shame due to their work in the sex industry which resulted in them being unwilling to disclose their work to friends and family. As found in this study and others, the shame associated with doing sex work contributed to many women not disclosing the nature of their work for fear of being judged or rejected [14, 17–19].

It was also common for women in this study to feel the need to maintain a distinction between their work and personal life, using separation as a coping mechanism to manage the two spheres of their lives, including not socialising with other sex workers, and using condoms with clients but not with romantic partners. This has previously been suggested to reflect levels of intimacy in relationships as well as creating a symbolic barrier between sex at work and sex at home [34, 37]. Other common coping mechanisms sex workers use to separate the two spheres, a number of which were identified in this study, include lying to their partners and significant people in their lives about their work, trying to maintain a psychological distinction between sex at work and home, and changing dress, makeup and even persona in order to maintain distinctions between their work life and personal life [19, 25–29, 37]. It is likely these coping mechanisms are used not only to separate sex at work from sex at home, but as a means of maintaining sex workers’ emotional wellbeing. The stigma associated with sex work is likely to prevent women from being able to breakdown the borders between their work and personal lives, particularly where partners are not supportive or understanding of the nature of their work which contributes to their inability to discuss their work openly.

The theory of mentally separating work and home has been previously explored through the lens of border theory which posits that when work and home lives are very different it is important to maintain strong borders around them in order to lead a balanced life [34]. The women in this study appeared to have mixed reactions around mentally separating their work and home life, with the majority of women finding it useful to maintain a distinction between the two, and the few who felt it was unnecessary more likely to view sex work as an important part of their lives and identity. Previous research has similarly shown that creating distinctions between work and personal lives was an important aspect of coping for many women in the sex industry [17, 32, 45]. The ability to do this can depend on individual differences such as personal coping style and ways of thinking about their work. Some women found separating the two worlds useful and even had a separate persona for work than for home as has been shown previously [17, 19, 29, 32]. Women who viewed sex work as part of their lives and who they were, were more likely to be in a position to freely discuss their work with their romantic partners, most of who accepted it well and often had a greater understanding of the industry. Partners’ greater understanding and acceptance of the nature of their work meant that women often felt less of a need to separate their two worlds and the boundaries between these worlds were more permeable [34]. Women who had supportive partners tended to report more positive experiences of the impact of work on their relationships and demonstrated a more integrated psychological approach to work and home life balance. According to border theory, spouses tend to have a degree of control over their partner’s balance between work and home and the borders between the two. This is exemplified in partners reactions to sex work and the effects this had on sex workers’ experiences of balancing the two worlds [34].

Interestingly, single women in this study commonly chose not to have a relationship while working in the sex industry for the same reasons the women in relationships raised. Women did not want to have to lie to potential partners or deal with the trust issues they knew would inevitably arise. These findings are consistent with previous study findings by Warr and Pyett [37], who reported that a number of women were concerned about having a relationship while working in the sex industry for these reasons. As we found in this study, a considerable number of women also reported they did not want a relationship while working in the sex industry as the relationships available to them did not seem to fit with their idea of a healthy relationship. Women reported that they did not want a partner who would be comfortable with them doing sex work and associated this with commitment, respect and love. This relationship paradox whereby women felt it was impossible to have a relationship while working in the sex industry as it would only be possible with a man that they would not want to be with is worth exploring further. While the women themselves may be comfortable with their choice to work in the sex industry they do not want a partner who is comfortable with them engaging in sex work, indicating their views of sex work may be much more complex than is initially apparent, and they may not be as comfortable with sex work as it appears.

### Strengths and limitations

The major strength of this study is that it adds to the very limited data available on the impact of sex work on women’s personal intimate relationships. To our knowledge this is the first study to specifically explore the experiences of indoor sex workers in relation to the impact of sex work on their personal relationships and the use of mental separation as a coping mechanism. Previous studies have focused mainly on sex workers’ use of and reasons for using condoms at work compared to at home, and the associations women have with condom use at work and in their personal lives. A further strength of this study is that it focused on sex workers who are involved in the legal sex industry where occupational health and safety regulations are enforced. Women are more likely to present with issues due to the work itself, such as issues regarding their emotional wellbeing and relationships, rather than, for example, issues around personal safety. Although indoor sex workers safety is still of some concern it is much more likely to be an issue in the illegal sex industry. The qualitative component of the analysis allowed for an exploration of the breadth of women’s experiences and relationships which are diverse. This study also investigated single women’s views of sex work and relationships which has not been focused on in prior research.

The study had a number of limitations. Firstly, the results of this study are based on a relatively small sample of indoor sex workers from one sexual health centre in Victoria, Australia and as such the findings may not be generalizable to the broader population of sex workers in Australia. The purpose of this study however, was not to provide generalizable data but rather to conduct a preliminary examination in an area of little research to gain further insight and identify key issues relating to the impact of sex work on women’s personal romantic relationships. We have been successful in identifying a number of avenues that are important for further investigation and future large scale studies among a broad, diverse sample of sex workers are now required to confirm the findings of this study and determine generalisability. Secondly, the depth of data collection was not at the level of an interview style qualitative study. The self-report nature of the questionnaire may not have allowed women to fully explore their feelings and experiences in the open text areas, however, the anonymous nature of the questionnaire may have also allowed women to feel freer to express their feelings and opinions more honestly without the presence of an interviewer. The self-report method may also have limited the findings due to potential responder bias however, again, it is possible that in being anonymous women may have been more comfortable and honest about their experiences than if they were identifiable or the questionnaire was interviewer administered. Lastly, due to constraints on nurses’ time during consultations we were also unable to keep an accurate record of the number of women who were handed out a questionnaire and as such are unable to provide an accurate participation rate.

### Implications and further research

This exploratory study identified some key issues women working in the sex industry face when trying to balance their work and personal romantic relationships. This study enabled women to share some of the emotional impacts of their work, the information of which is likely to be useful to health care and support workers in assisting sex workers to manage the tensions between their work and personal romantic relationships. While these findings are clearly not generalizable to the wider community of sex workers, they have provided a useful insight into this largely under researched area, and support the need for a larger study to be undertaken to determine if the findings of this study are reflected in a larger, more representative sample of Australian sex workers. Consideration should be given to including both indoor and outdoor sex workers who face considerably different work and personal issues which are likely to impact on their personal romantic relationships in different ways. It is likely women from different socio-economic and cultural backgrounds, diverse sexualities and partner type, and geographic area will experience differing impacts of sex work and it is important future interventions recognise and tailor support programs accordingly. It is also imperative, given the significant barrier it plays in most women’s relationships, that the negative stigma surrounding the sex industry is addressed through greater public awareness and education campaigns. It is possible other associated issues faced by women such as dishonestly and lying would be of less concern if they felt confident and comfortable to disclose their true profession to partners, family and friends without fear of judgement or stigmatisation. Nevertheless, the issues that women face in their relationships as a result of sex work are clearly complex and there will not be one simple solution to address such a wide range of experiences.

## Conclusions

The findings of the current study suggest that sex work impacts personal romantic relationships in mainly negative ways. The impacts ranged in manifestation and severity but overwhelmingly caused issues around trust, deception, lying and jealousy. Negotiating the viability of potential relationships while working in the sex industry was an issue for a variety of reasons including stigma, trust and the types of relationships that women felt they wanted. It is important to note however, that a minority of women did report positive effects of sex work on their relationships and sex lives, which highlights the diversity of experiences in this group of women.

## References

[1] Burnes TR, Long SL, Schept RA. A Resilience-Based Lens of Sex Work: Implications for Professional Psychologists. Professional Psychology: Research and Practice. 2012;43(2):137–44.

[2] Bernstein E. What's Wrong with Prostitution? What's Right with Sex Work? Comparing Markets in Female Sexual Labor. Hastings Women's Law Journal. 1999;10(1):91–117.

[3] Shaver FM. Sex Work Research: Methodological and Ethical Challenges. Journal of Interpersonal Violence. 2005;20(3):296–319. 15684139 10.1177/0886260504274340

[4] Schampheleire DD. MMPI Characteristics of Professional Prostitutes: A Cross-Cultural Replication. Journal of Personality Assessment. 1990;54(1&2):343–50. 2313550 10.1080/00223891.1990.9673998

[5] McClanahan SF, McClelland GM, Abram KM, Teplin LA. Pathways into Prostitution Among Female Jail Detainees and their Implications for Mental Health Services. Psychiatric Services. 1999;50(12):1606–13. 10577881 10.1176/ps.50.12.1606

[6] Cusick L. Youth Prostitution: A Literature Review. Child Abuse Review. 2002;11:230–51.

[7] Cusick L. Widening the Harm Reduction Agenda: From Drug Use to Sex Work. International Journal of Drug Policy. 2006;17:3–11.

[8] Rekart ML. Sex-Work Harm Reduction. Lancet. 2005;366:2123–34. 16360791 10.1016/S0140-6736(05)67732-X

[9] O'Doherty T. Victimisation in Off-Street Sex Industry Work. Violence Against Women. 2011:1–20. 10.1177/1077801211412917 21665856

[10] Surratt HL, Kurtz SP, Chen M, Mooss A. HIV Risk Among Female Sex Workers in Miami: The Impact of Violent Victimisation and Untreated Mental Illness. AIDS Care. 2012;24(5):553–61. doi: 10.1080/09540121.2011.630342 22085330 10.1080/09540121.2011.630342 PMC3342480

[11] Farley M, Barkan H. Prostitution, Violence Against Women, and Posttraumatic Stress Disorder. Women and Health. 1998;27(3):37–49. 9698636 10.1300/J013v27n03_03

[12] Surratt HL, Kurtz SP, Weaver JC, Iniardi JA. The Connections of Mental Health Problems, Violent Life Experiences, and the Social Milieu of the 'Stroll' with the HIV Risk Behaviours of Female Street Sex Workers. Journal of Psychology and Human Sexuality. 2005;17(1):23–44.

[13] Cwikel J, Ilan K, Chudakov B. Women Brothel Workers and Occupational Health Risks. Journal of Epidemiological Community Health. 2003;57:809–15. 10.1136/jech.57.10.809 PMC1732308 14573588

[14] Scrambler G. Sex Work Stigma: Opportunist Migrants in London. Sociology. 2007;41(6):1079–96.

[15] Scrambler G, Paoli F. Health Work, Female Sex Workers and HIV/AIDS: Global and Local Dimensions of Stigma and Deviance as Barriers to Effective Interventions. Social Science & Medicine. 2008;66:1848–62. 18295948 10.1016/j.socscimed.2008.01.002

[16] Cornish F. Challenging the Stigma of Sex Work in India: Matierial Context and Symbolic Change. Journal of Community and Applied Social Psychology. 2006;16(6):462–71.

[17] Fick N. Coping with Stigma, Discrimination and Violence: Sex Workers Talk about their Experiences. In: Force SWEaAT, editor. Cape Town: SWEAT; 2005 p. 1–65.

[18] Murphy AK, Venkatesh SA. Vice Careers: The Changing Contours of Sex Work in New York City. Qualitative Sociology. 2006;29:129–54.

[19] Sanders T. 'It's Just Acting': Sex Workers' Strategies for Capitalising on Sexuality. Gender, Work and Organisation. 2005;12(4):319–41.

[20] Quadara A. Sex Workers and Sexual Assault in Australia, Prevalence, Risk and Safety. In: Assault ACftSoS, editor. 2008.

[21] Prostitution Control Act. State of Victoria1994.

[22] Public Health and Wellbeing Act. In: Victoria So, editor. 2008.

[23] Woodward C, Fischer J, Najman J, Dunne M. Selling Sex in Queensland. In: Authority PL, editor. Queensland 2003.

[24] Donovan B, Harcourt C, Egger S, Smith LW, Schneider K, Kaldor J, et al The Sex Industry in New South Wales: a Report to the NSW Ministry of Health. In: Wales UoNS, editor. Sydney: The Kirby Institute; 2012.

[25] Bilardi JE, Miller A, Hocking JS, Keogh L, Cummings R, Chen MY, et al The Job Satisfaction of Female Sex Workers in Licensed Brothels in Victoria Australia. Journal of Sexual Medicine. 2011;8:116–22. doi: 10.1111/j.1743-6109.2010.01967.x 20722786 10.1111/j.1743-6109.2010.01967.x

[26] Dalla RL, Xia Y, Kennedy H. "Just Give them what they Want and Pray they Don't Kill You": Street-Level Sex Workers' Reports of Victimization, Personal Resources, and Coping Strategies. Violence Against Women. 2003;9(11):1367–94.

[27] Murray L, Moreno L, Rosario S, Ellen J, Sweat M, Kerrigan D. The Role of Relationships Intimacy in Consistent Condom Use Among Female Sex Workers and their Regular Paying Partners in the Dominican Republic. AIDS Behavoiur. 2006;11:463–70. 10.1007/s10461-006-9184-5 17096198

[28] Sanders T. The Condom as Psychological Barrier: Female Sex Workers and Emotional Management. Feminism and Psychology. 2002;12(4):561–6.

[29] Wolffers I, Triyoga RS, Basuki E, Yudhi D, Deville W, Hargono R. Pacar and Tamu: Indonesian Women Sex Workers' Relationships with Men. Culture, Health and Sexuality. 1999;1(1):39–53. 12295114 10.1080/136910599301157

[30] Rhodes T, Cusick L. Love and Intimacy in Relationship Risk Management: HIV Positive People and their Sexual Partners. Sociology of Health and Illness. 2000;22(1):1–26.

[31] Warr DJ, Pyett PM. Difficult Relations: Sex Work, Love and Intimacy. Sociology of Health and Illness. 1999;21(3):290–309.

[32] Sanders T. A Continuum of Risk? The Management of Health, Physical and Emotional Risks by Female Sex Workers. Sociology of Health and Illness. 2004;26(5):557–74. 15283777 10.1111/j.0141-9889.2004.00405.x

[33] Rossler W, Koch U, Lauber C, Hass A-K, Altwegg M, Ajdacic-Gross V, et al The Mental Health of Female Sex Workers. ACTA Psychiatrica Scandanavica. 2010:1–10. 10.1111/j.1600-0447.2009.01533.x 20105147

[34] Clark SC. Work/Family Border Theory: A New Theory of Work/Family Balance. Human Relations. 2000;53(6):747–70.

[35] Brody S, Potterat JJ, Muth SQ, Woodhouse D. Psychiatric and Characterological Factors Relevant to Excess Mortality in a Long-Term Cohort of Prostitute Women. Journal of Sex and Marital Therapy. 2005;31:97–112. 15859370 10.1080/00926230590477943

[36] Vanwesenbeeck I. Burnout Among Female Indoor Sex Workers. Archives of Sexual Behaviour. 2005;34(6):627–39. 10.1007/s10508-005-7912-y 16362247

[37] Warr DJ, Pyett PM. Difficult Relations: Sex Work, Love and Intimacy. Sociology of Health & Illness. 1999;21(3):290–309.

[38] Stebbins R. Exploratory Research in the SOcial Sciences. CA: Sage; 2001.

[39] Creswell J. Research Design: Qualitative, Quantitative, and Mixed Methods Approaches. California: Sage Publications; 2003.

[40] Goodman LA, Corcoran C, Turner K, Yuan N, Green BL. Assessing Traumatic Event Exposure: General Issues and Preliminary Findings for the Stressful Life Events Screening Questionnaire. Journal of Traumatic Stress. 1998;11(3):521–42. 9690191 10.1023/A:1024456713321

[41] Bellavia G, Frone M. Work-Family Conflict Handbook of Work Stress 2005 p. 113–47.

[42] LeCompte MD, Schensul JJ. Designing and conducting ethnographic research: AltaMira Press; 1999 240 p.

[43] Braun V, Clarke V. Using Thematic Analysis in Psychology. Qualitative Research in Psychology. 2006;3(2):77–101.

[44] Yperen NWV, Buunk BP. A Longitudinal Study of Equity and Satisfaction in Intimate Relationships. European Journal of Social Psychology. 1990;20:287–309.

[45] Sanders T. Becoming an Ex-Sex Worker: Making Transitions out of a Deviant Career. Feminist Criminology. 2007;2:74–95.

